# Assessment of viscoelasticity and hydration effect of herbal moisturizers using bioengineering techniques

**DOI:** 10.4103/0973-1296.71797

**Published:** 2010

**Authors:** Shweta Kapoor, Swarnlata Saraf

**Affiliations:** *Institute of Pharmacy, Pt. Ravishankar Shukla University, Raipur (C.G), 492010, India*

**Keywords:** Bioengineering techniques, herbal moisturizer, hydration, viscoelasticity

## Abstract

**Background:**

A number of moisturizers are available containing natural hydrating, moisturizing, fi rming and occlusive property-imparting agent in the form of herbal extracts, juice and oils. The aim of this study is to assess the hydration and viscoelastic effect of commercially available herbal moisturizers, containing different herbs, on human skin, after a single and 3-week period of application using skin bio mechanical and electrical techniques.

**Materials and Methods:**

Twenty selected herbal moisturizers (HM) were coded as HM1-HM20. Forty volunteers, mean age of 40 ± 9 years, were participated in the short- and long-term study. Skin properties in terms of hydration and viscoelastic parameters were measured by multitester and cutometer, respectively. Measurements were done before and after 1, 2, and 3 h (single application) and for the 3-week period of daily application.

**Results:**

After single application, significant increase has been observed in both the skin electrical (*P* < 0.001) and mechanical properties (*P* < 0.01) as compared to the control, at which no products were applied. After the 3-week period, both effects are maintained and found to be significant at *P* < 0.001. Short-and long-term study revealed that out of 20 herbal moisturizers, HM8 and HM10 show pronounced increase in skin hydration (90-100%) and HM8, HM10, and HM11 shown marked increase in skin viscoelasticity (90-95%).

**Conclsuion:**

The possible reason of maximum effects obtained by these products is multifunctional effects of active ingredients of incorporated herbs. Combined used of both non invasive techniques is useful to substantiate the hydrating and viscoelasticity claim of herbal moisturizer. Short- and long-terms study revealed the best performing herbal moisturizer.

## INTRODUCTION

The appearance and function of the skin are maintained by an important balance between the water content of the stratum corneum and skin surface lipids.[1,2] Exposure to external factors such as air humidity, ultraviolet radiation, temperature, as well as endogenous factors, i.e., hormones,[3–5] may disrupt this balance. In addition, frequent use of soaps, detergents, and topical irritants such as alcohol and hot water can remove the skin surface lipids.[6] When this balance is disrupted, skin mechanical properties and water content get disturbed; hence, skin becomes dry and loses its elasticity. In these cases, effective dermatocosmetic products must be used to improve the skin hydration and viscoelasticity not only for aesthetic purposes but also to maintain the normal conditions of skin and to prevent dry skin alterations.[7] The moisturizing effect of formulations may be influenced by many factors, such as type and concentration of the active substances used, as well as the composition of the vehicle.[8,9]

Moisturizers are also the most prescribed products in dermatology.[10] A number of herbal moisturizers are floating in the market with effective claims under the bandwagon of naturals. Most of the commercial moisturizer contains *Aloe barbadensis* (Aloe vera) as a moisturizing agent.[11] Nowadays there so many other herb’s extract/juices/oils like grape seed, cucumber, basil, jojoba oil, almond oil, olive oil, etc. present in the commercial moisturizer section claiming for restoring skin hydration and viscoelasticity properties. Consequently, subjective studies to evaluate the moisturizing effect scientifically are necessary to validate these claimed effects. Objective methodologies are considered appropriate to prove and to clarify the mechanisms of action of substances that improve skin properties.

Several *in vivo* studies using modern bioengineering techniques have been performed to evaluate the mechanical properties and water content of the epidermis.[12–15] Instruments that have been used for assessing epidermal hydration are based on measurements of conductance,[16] capacitance,[12,17–19] and impedance[20] of the skin. Instruments that have been used for testing skin (viscoelasticity) mechanical properties are based on torsion[21] and suction methods.[12–21] Proof of efficacy can be evaluated in different ways[22] but testing on human volunteers is the preferred one. By introducing noninvasive instrumental measuring techniques that are applicable on humans, properties of cosmetic and dermatologic products can be assessed in an objective way, making comparisons between results possible and allowing the detection of subtle changes in skin morphology and function otherwise not detectable by sensorial means. The objective of research in herbal cosmetic field is obtaining the best from the nature for better tomorrow.[23] In the present research, noninvasive skin biomechanical and electrical techniques have been used to evaluate the viscoelastic and hydration effects of 20 commercial herbal moisturizers containing different moisturizing actives in the form of herbal ingredients; this assessment would provide support in establishing a strong faith of consumer towards herbal moisturizers.

## MATERIALS AND METHODS

### Products

Ttwenty commercially available herbal moisturizers were purchased from local cosmetic dealer at Raipur, Chhattisgarh, India. Out of them, 8 were products from leading abroad manufacturers and 12 were products of top most Indian manufacturers. The products were referred by the codes HM1–HM20. Base ingredients that were indicated on the packages of selected moisturizers are summarized in Table 1. Other qualitative ingredients mainly herbal extracts, juices, and oils vary product to product and are listed in Table 2.

**Table 1:** List of base ingredients in herbal moisturizers

Herbal moisturizer	Common base ingredients
HM1–HM20	Aqua, paraffinium, glycerin, butylene glycol, alcholol denatured, stearic acid, glyceryl stearate, coco glycerides, dimethicone, carbomer, TEA, NAdiEDTA lanolin, methylparaben, butylp, ethylp, propylp, parfum.

**Table 2:** List of other ingredients present in herbal moisturizers

Herbal moisturizer	Ingredients
HM1	Jojoba, vit E
HM2	*Chamomilla recutita, Helanthus annuus, Sambucus nigra, Primula veris, Theobroma cacao*
HM3	Hydolyzed elastin, Talc, Tocopheryl acetate
HM4	Aloe barbadensis
HM5	*Elaeis guineensis, Olea europaea, Persa fratissima, Prunus armeniaca, Ribes nigrum, Vitis vinifera*, Micro fruit oil
HM6	Shea butter, *Cocos nucifera, Olea europaea* fruit oil (Olive), *Aloe barbadensis* (leaf)
HM7	Vit E, Vit A, *Theobroma cacao*, Pollen extract, *Triticum vulgare* (Wheat germ oil)
HM8	*Cucumis sativus* juice, Coumarin, Hexyl cinnamal, Limonene
HM9	Aloe vera, Indian madder, Country mallow,
HM10	Kapoor kachari, Chandan, Nimba, Ghrit kumari, Ushir, Gulabjal, Tulasi, Haridra, Yastimadhu, Malai, Grape seed oil, Olive oil, Badam oil, Keshar, Bhavpralash, Tankan amla (Boric acid), Rastarangni
HM11	Santalum album (Sandal wood), Cuscus grass (Vetiveria zizanioides), Sweet basil (Ocimum sanctum), Aloe vera, Honey
HM12	Behda Kwath, Madhu, Ankurit gehum, Kusumbhi tail, Methi beej, Vach
HM13	Olive oil, Sesame oil, Vit E
HM14	Olive oil, Red apple
HM15	Aloe vera, Jojoba oil, Milk cream, Wheat germ
HM16	Vit A, D, E, Aloe vera, Wheat germ oil, Rose water
HM17	Almond, Sandal wood, Honey, Wheat Germ oil, Jojoba oil, Essential oil of patchouli, Germanium, Rose and Basil
HM18	Grape seed, Wheat germ oil, Vit E, Vit F
HM19	Cocoa butter, Vit E, Aloe vera ext
HM20	Honey, Almond

### Subjects and study protocol

A total of 40 healthy volunteers, mean age 40 ±9 years, were studied after they provided informed consent. A dermatologist determined the skin types of these volunteers as normal to dry. All participating volunteers were found free from any pathological findings on their arms. All test subjects were asked not to use cleansing or skin care products on the volar forearms[24] for 1 week prior to and during the study. Viscoelastic and hydration measurements were carried out (baseline) before and after the application of the moisturizers (short-term test; 1, 2, and 3h) after a single application. This procedure was followed for a long-term test of 3 weeks, wherein herbal moisturizers were applied 2 times daily in the morning and evening for 20 days consecutively. About 0.2 g of each moisturizer was applied to different test sites, 2 cm in diameter located on the volar forearm. An adjacent untreated skin area served as a control. Cutometer and conductance measurements were then performed 12 h after the last application, i.e, day 21. The measurements were performed in an acclimatized room with a mean relative humidity of 40 ± 3% and a mean room temperature of 23 ± 5°C. They were carried out under standardized conditions as described in earlier[25] literatures. All measurements were carried out according to the relevant guidelines,[26] nontreated sites on volar forearm served as controls.

### Techniques

#### Cutometer

**Figure 1:**
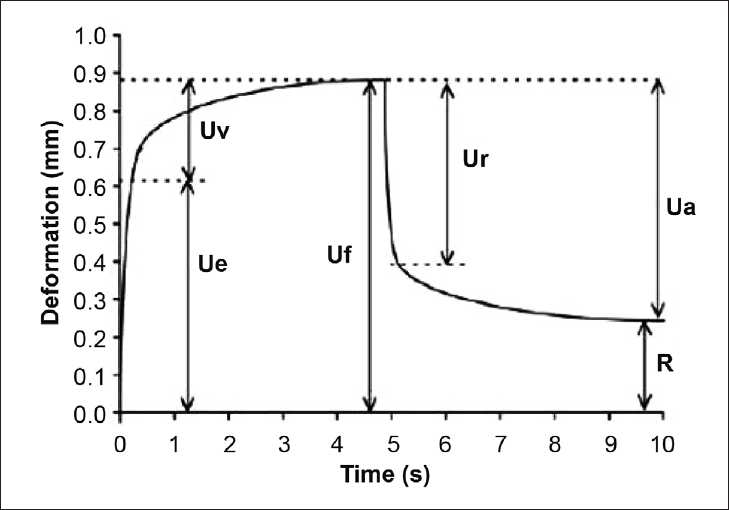
Skin deformation curve obtained with Cutometer

The mechanical properties of the epidermis were determined using a noninvasive, *in vivo* suction skin elasticity meter, Cutometer (MPA 580, Courage and Khazaka, Koln, Germany) equipped with 2 mm measuring probe. The time/strain mode was used with a 5 s application of a constant negative pressure of 500 m bar, followed by a 5-s relaxation period. A typical skin deformation curve is illustrated in Figure 1. The following parameters were analyzed: *Ue*, immediate distension; *Uv*, delayed distension; (R_0_) *Uf*, final distension (skin distensibility); *Ur*, immediate retraction; R, residual deformation at the end of measuring cycle (resilient distension); (R_2_) *Ua/Uf*, gross-elasticity of the skin, including viscous deformation; (R_5_) *Ur/Ue*, neto-elasticity of the skin without viscous deformation; (R_7_) *Ur/Uf*, biological elasticity, i.e., the ratio of immediate retraction to total distension; (R_6_) *Uv/Ue*, the ratio of viscoelastic to elastic distension; and R8, viscopart, i.e., the area under the suction part of the deformation curve. The average values of two measurements were used in subsequent calculations. The curves of the obtained skin deformation values were analyzed using the software of Cutometer MPA 580.

#### Multitester

Hydration of the epidermis was determined with a noninvasive technique using an electronic device, Multitester (CASIO, H-21, India), which measured resistance, based on the commonly known fact that hydrated skin has less resistance to current flow than dehydrated skin. The level of stratum corneum hydration was assessed by measurement of the changes in skin resistance and is referred to as the galvanic skin response or electrical skin resistance. The skin resistance reported in ohms with electrodes (size 1 cm ^2^) was measured after 1, 2, and 3 h and then daily upto 3 weeks for long term study, after application of each moisturizer, at 1000 khz, 10 mA, AC current according to the modification of Nicander *et al*.[27,28]

### Statistical Analysis

Statistical analysis was carried out by using the STAT software, obtained values were expressed as mean ± SD (standard deviation). Viscoelasticity and hydration values of the tested sites were expressed as a percentage of the values obtained for the appropriate control site on volar forearm arbitrarily set to 100%. Analysis of variance (ANOVA) and Student’s paired *t*-tests were performed. Differences was considered statistically significant if *P* < 0.01 and highly significant if *P* < 0.001.

## RESULTS

### After short-term study

**Figure 2:**
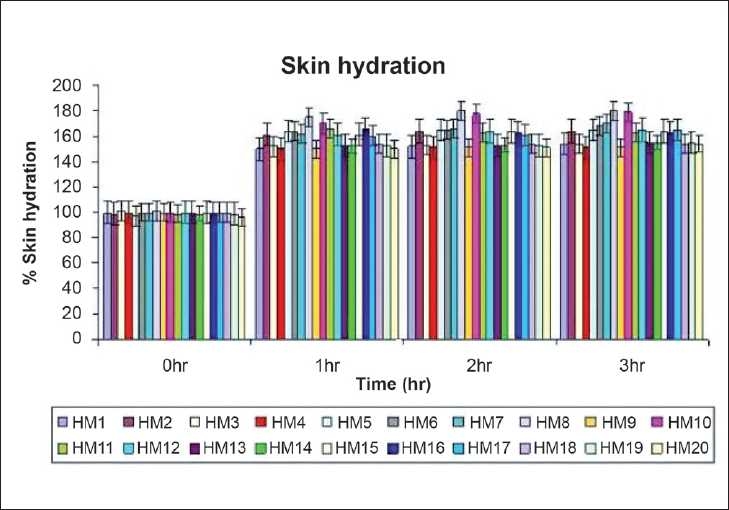
Changes in % of skin hydration measured before (baseline) and after 1, 2 and 3hr of single application of herbal moisturizers

Electrical measurements by multitester in terms of hydration obtained during short-term study are reported in Table 3. Highly significant increases in the water content of stratum corneum readings (*P* < 0.01) relative to baseline (before, 0 h) were observed 1, 2, and 3 h after single application of all moisturizers [Figure 2]. After 3 h of single application of moisturizers, HM8 and HM10 has shown 70–80% increase in water content of stratum corneum, HM2,5,6,7,11,12,15,17, and 16 has shown 60–70% increase, and 16 has shown 60–70% increase, and 50–55% increase was shown by HM1,3,4,9,13,14,18, and 19 when compared to baseline (control) reading that is set to an arbitrary value [Figure 2].

**Figure 3:**
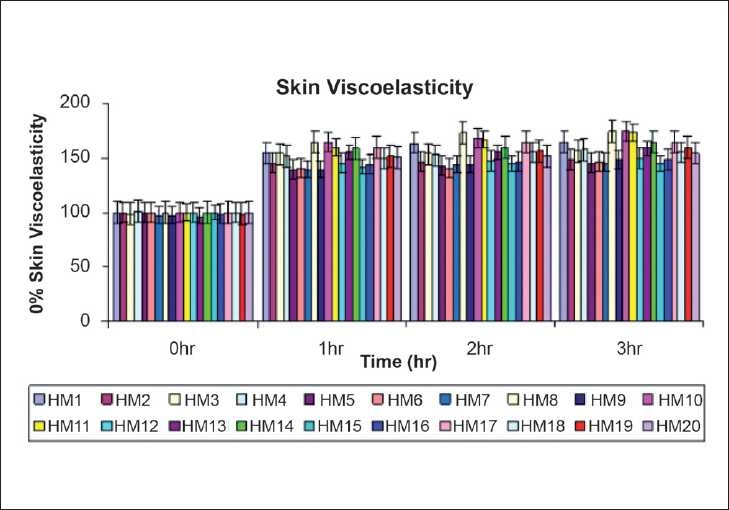
Changes in % of skin viscoelasticity measured before (baseline) and after 1,2 and 3 hr of single application of herbal moisturizers

The changes in skin mechanical parameters observed before and after 1, 2, and 3 h after single application of moisturizers is expressed as a percentage increase in the values and are shown in Table 3. Significant increase (*P* < 0.01) in viscoelastic parameters, relative to baseline (before, 0 h) were observed 1, 2, and 3 h after single application of all moisturizers [Figure 3]. The most pronounced changes were observed with moisturizer HM8, HM10, and HM11 (65–75% increase), compared with HM1,3,4,13,14,17,18,19, and 20 (60–55% increase) and 40–50% increase was shown with HM2,5,6,9,7,12,15, and 16 when compared to baseline [Figure 3].

**Table 3:** Skin hydration and viscoelasticity after 3 h (short-term study)

Moisturizers	% increase in skin hydration			% increase in skin viscoelasticity		
	1 h	2 h	3 h	1 h	2 h	3 h
HM1	150±9	152±8	154±8	155±10	164±9	165±10
HM2	151±8	152±9	152±9	145±9	147±9	149±8
HM3	152±7	153±8	154±7	154±7	154±7	157±10
HM4	150±9	151±9	151±8	152±10	153±10	158±9
HM5	151±8	153±7	155±8	140±9	143±9	145±8
HM6	153±7	153±6	154±6	141±9	141±7	147±7
HM7	152±7	152±7	152±5	140±8	144±7	146±8
HM8	175±6	180±8	180±7	165±9	174±10	175±10
HM9	170±7	178±7	179±7	140±7	144±7	149±8
HM10	165±6	170±8	171±6	165±9	168±9	175±8
HM11	161±7	164±6	165±6	160±8	167±8	174±7
HM12	162±6	162±9	164±8	145±7	148±9	150±9
HM13	160±7	161±9	165±8	156±7	156±7	159±7
HM14	166±6	163±6	163±6	159±8	160±9	165±10
HM15	161±9	164±8	164±9	142±7	145±7	145±7
HM16	164±8	165±7	168±7	144±8	147±8	149±9
HM17	162±8	166±6	170±6	160±7	165±10	165±10
HM18	161±7	164±7	165±8	150±8	156±9	156±9
HM19	163±8	165±8	170±9	152±9	157±7	160±10
HM20	165±7	165±7	169±6	151±10	152±9	155±9

Mean ± SD (Standard deviation), *P* < 0.01

### After long-term study

**Figure 4:**
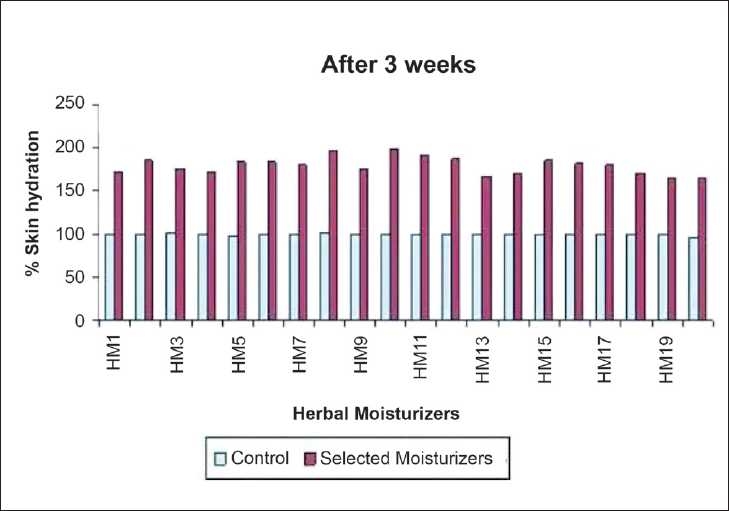
Changes in skin hydration after 3 week period of daily application of selected herbal moisturizers expressed as a percentage increase as compare to control arbitrarily to 100%

All participants reported strict compliance with the instructions. Effect after 3 weeks of daily applications of moisturizers was measured by mutlitester and is reported in Table 4. The results obtained after a 3-week period application shown that all moisturizers produced a significant increase (*P* < 0.001) in skin hydration when compared with baseline [Figure 4]. However, when these moisturizers were compared with each other, the maximum percentage increase in skin hydration was obtained with HM8 and HM10 (95–100%). During study HM2,5,6,7,11,12,15,17,16 has shown 80-90% increase and HM1,3,4,9,13,14,18,19 has shown 60–70% increase in skin hydration after 3 weeks when compared to baseline reading.

**Figure 5:**
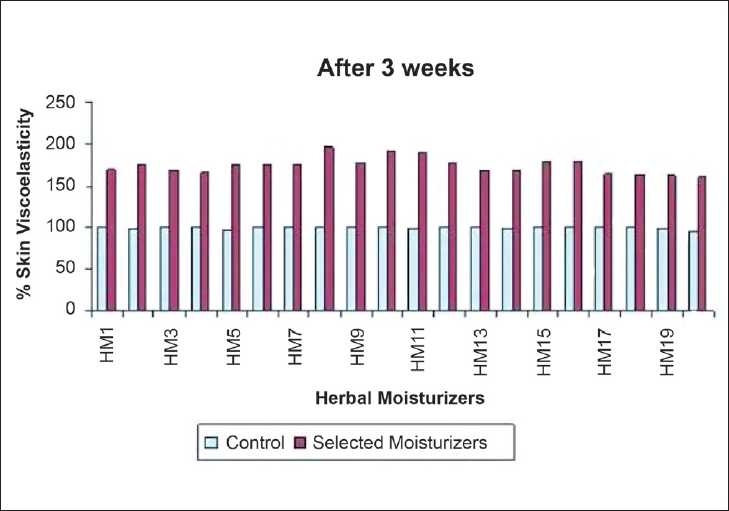
Changes in skin viscoelasticity after 3 week period of daily application of selected herbal moisturizers expressed as a percentage increase as compare to control arbitrarily set to 100%

The changes in skin mechanical parameters observed after 3 weeks of daily application of moisturizers is expressed as a percentage increase in the values and is shown in Table 4. The results obtained after 3 weeks were significantly higher (*P* < 0.001) relative to baseline reading [Figure 5]. The most pronounced changes were observed with moisturizer HM8, HM10, and HM11 (90–95% increase), then with HM1,3,4,13,14,17,18,19, and 20 (75-80% increase) and 60-70% increase with HM2,5,6,9,7,12,15, and 16 when compared to control [Figure 5].

**Table 4:** Increase in skin hydration and viscoelasticity after 3 weeks (long-term study)

Moisturizers	Skin hydration (%)	Viscoelasticity (%)
HM1	172±4	169±9
HM2	185±7	176±6
HM3	175±6	167±8
HM4	171±7	166±8
HM5	183±6	175±7
HM6	184±7	176±9
HM7	180±3	175±6
HM8	197±7	195±9
HM9	175±6	177±7
HM10	199±6	193±8
HM11	190±5	190±7
HM12	187±4	177±9
HM13	166±4	168±6
HM14	169±4	167±9
HM15	186±5	180±9
HM16	182±4	179±7
HM17	180±4	165±6
HM18	170±5	162±8
HM19	164±6	162±7
HM20	165±7	160±8

Mean ± SD (Standard deviation), *P* < 0.001

## DISCUSSION

Effects of various herbal moisturizers on skin bio mechanical and electrical have been observed in terms of short term and long term studies. Skin hydration and viscoelastic measurements are carried before and after 1, 2, and 3 h of application of the product, as it is possible to attain improved skin properties shortly after a single application.[29] Nevertheless, long-term studies (3 weeks) are important to assess the maintenance and enhancement of these effects. The short-term test allows in most cases high discrimination, whereas the long-term test usually shows the real effects the products have on the upper layers of the skin when the product is no longer present.[26,30] In both types of test, it is advisable to work with a target group of elderly volunteers as epidermal properties has seen more altered for such persons. For these reasons, we have studied the effect of hydrating and viscoelastic properties of herbal moisturizers in elderly people. In the present study, we adopted a combination of noninvasive bio mechanical and electrical techniques for the measurement of skin hydration and viscoelasticity, respectively.

Interpretation of results revealed that there are significant increases in skin hydration and viscoelastic parameters after single application of all herbal moisturizers, which are further increased and maintained after regular application of them upto 3 weeks. Results of our study are in agreement with the report by Li *et al*.[9] The increase in water content and viscoelasticity could, however, be due to different reasons[31] associated with various herbal ingredients incorporated in selected moisturizers [Table 2]. Almost all the herbal moisturizer contain aloe vera (Ghrit kumari) extract, which is rich composition in hygroscope mono and polysaccharides[32] and in the amino acids, which may improve water retention in the stratum corneum.[33] The silica in cucumber (*Cucumis sativum*) is an essential component of healthy connective tissue, which includes muscles, tendons, ligaments, cartilage, and bone. It is an excellent source of potassium, vitamin C, and folic acid. The high water content makes cucumbers good for moisturizing effect. Methi (*Trigonella Foenum Graecum*) seed extract contains 45–60% carbohydrates, 5–10% fixed oils (lipids), flavonoids, free amino acids that provide softening, cleansing, soothing properties to skin. Sandalwood (Santalum Alba) the main constituent of sandalwood oil is santalol, credited for its moisturizing and viscoleastictity property. It has been used since earliest times as incense, in embalming and cosmetics.[33] Almond oil (*Prunus Amygdalus*) contains folic acid, alpha tocopherol, and zinc, which are useful in skin disorders.[34] Wheat germ oil (*Triticum sativum*); wheat is a rich source of tocopherols with high vitamin-E potency that nourishes and prevents loss of moisture from the skin. Red apple (*Pyrus malus*); it is a rich source of various vitamins, trace elements, amino acids, and flavanoids due to which it acts as humectant and provide moisturizing and viscoelasticity property. Coconut (*Cocos Nucifera*) oil helps keep skin soft and smooth. Lauric oils, the dominant fatty acid (45–48%) in coconut oil, are used in cosmetics.[35] Yashtimadhu (*Glycyrrhiza Glabra*) extract is helpful to formulate cosmetic products for the protection of skin and hair against oxidative processes.[36] Grape seed (*Vitis vinifera*) contain pycogeneol,[37] which is responsible for its cosmetic properties. Many traditional herbs have been scientifically evaluated for their cosmetic potential[38] like Olive oil (*Oleum olivae/ Olea europaea*), Neem (*Azadirachta indica*), Tulsi (*Ocimum santum*), Geranium (*Pelargonium graveolen*), Kapoor Kachari (*Kaempferia galanga*), Cuscus grass th (*Vetivera Zizanozides, Ushir*), Kesar (*Crocus sativus*), Khumani (*Prunus armeniaca*), Jojoba (*Simmondsia chinensis*), and Indian madder (*Rubia cordifolia*), etc.

All products shown high improvement in skin hydration but HM8 and M10 both gave highest hydration of the stratum corneum of elderly volunteers. The high hydration effect obtained by HM8 and HM10 is due to synergetic effect of various natural moisturizing herbs [Table 2].

The most pronounced changes in skin viscoelastic property were observed with HM8, 10, and 11 due to increase in *Uv* and *Uv/Ue* parameters. *Uv* and *Uv/Ue* represent the viscoelastic part of the skin deformation.[39,40] Increase in these parameters values indicates the decrease in the viscosity of the interstitial fluid as a result of the increased water content and changes in the proteoglycan composition and/or structure.[41] The accumulation of water in the dermis diminishes the friction between the fibres and facilitates the movement of the interstitial fluid. Our study also complies that there is a link between skin hydration and viscoelastic parameters (*Uv* and *Uv/Ue*). Out of 20 selected moisturizers the pronounced change in both properties of skin has been observed with HM8 and HM10. With HM11 skin hydration did not increase linearly with skin viscoelasticity, this may be due to its ingredient’s properties.

The present study emphasized on the moisturizing properties of the herbal products. Repeat applications of the formulations induced a higher level of stratum corneum moisturization as compared to single application. However, to maintain these effects, the products must be repeatedly applied because stopping the applications abates the benefit in stratum corneum hydration.

Study also indicates that the products which contains either herbal extract/seed/oil/juice/gel of aloevera, grape, almond, olive, wheatgerm, sandalwood, cucumber shown better viscoelastic and hydration effect as compared to other products.,

Thus, we suggest that the daily use of moisturizers containing herbal extract/juice/oils is important to maintain humectant and soothing effect on the skin. Results of noninvasive skin bioengineering techniques scientifically substantiate the hydrating and viscoelastic claims of commercially available herbal moisturizers.

## CONCLUSION

The main objective of this study was to evaluate and substantiate the viscoelastic and hydration effect of herbal moisturizers using noninvasive bio mechanical and electrical techniques. This study highlighted the influence of various herbal ingredients on the efficacy of cosmetic products. We conclude that epidermal hydration produced by moisturizers influences the mechanical properties of skin. Noninvasive skin mechanical and electrical measurements are appropriate for an objective and quantitative evaluation of the complex effect of different dermatological and herbal cosmetic products on epidermal mechanics and water content. Short- and long-term study contributes to select the best performing moisturizers and helps to elucidate the possible mechanism of action lying behind its use. This study can be helpful for upcoming researchers to select herbs for the formulation and evaluation of herbal moisturizers which can be claimed for their efficacy with scientific datas, which shall further give strength to our herbal and cosmetic industries eminence in global market.
